# Biological Effects of Shikonin in Human Gingival Fibroblasts via ERK 1/2 Signaling Pathway

**DOI:** 10.3390/molecules24193542

**Published:** 2019-09-30

**Authors:** Kazutaka Imai, Hirohito Kato, Yoichiro Taguchi, Makoto Umeda

**Affiliations:** Department of Periodontology, Osaka Dental University, 8-1, Kuzuhahanazonocho, Hirakata, Osaka 573-1121, Japan; imai-k@cc.osaka-dent.ac.jp (K.I.); kato-h@cc.osaka-dent.ac.jp (H.K.); umeda-m@cc.osaka-dent.ac.jp (M.U.)

**Keywords:** shikonin, wound healing, human gingival fibroblasts, ERK 1/2

## Abstract

Shikonin, an active ingredient of *Lithospermum erythrorhizon*, exerts anti-inflammatory and antibacterial effects, and promotes wound healing. We investigated whether shikonin stimulated gingival tissue wound healing in human gingival fibroblasts (hGF). In addition, we evaluated the effects of shikonin on the mitogen-activated protein kinase (MAPK) signaling pathway, which has an important role in wound healing. hGF were subjected to primary culture using gingiva collected from patients. The cells were exposed to/treated with Shikonin at concentrations ranging from 0.01 to 100 μM. The optimal concentration was determined by cell proliferation and migration assays. Type I collagen and fibronectin synthesis, the gene expression of vascular endothelial growth factor (VEGF) and FN, and the phosphorylation of Extracellular signal-regulated kinase (ERK) 1/2 were investigated. Identical experiments were performed in the presence of PD98059 our data suggest, a specific ERK 1/2 inhibitor. Shikonin significantly promoted hGF proliferation and migration. Shikonin (1 µM) was chosen as the optimal concentration. Shikonin promoted type I collagen and FN synthesis, increased VEGF and FN expression, and induced ERK 1/2 phosphorylation. These changes were partially suppressed by PD98059. In conclusion, Shikonin promoted the proliferation, migration, type I collagen and FN synthesis, and expression of VEGF and FN via ERK 1/2 signaling pathway in hGFs. Therefore, shikonin may promote periodontal tissue wound healing.

## 1. Introduction

Periodontal surgery is a common treatment for periodontal disease, and healing of the soft tissue is critical for the success of this therapeutic approach. However, infection at the wound site and excessive inflammation can delay wound healing. Immune cells (neutrophils, monocytes, lymphocytes, and dendritic cells), endothelial cells, keratinocytes, and fibroblasts are recruited to the wound site during wound healing [1,2]. In particular, human gingival fibroblasts (hGFs) play an important role in the cell growth and remodeling phases of oral wound healing in gingival tissue [3,4,5,6,7,8].

Naturally derived substances, including herbs, have been used as medicines for curing wounds for millennia, and have been studied extensively for their healing properties [9,10,11,12,13]. For example, the natural substances acemannan has been used to treat burns and skin wounds. Suwimon et al. demonstrated that acemannan induced type I collagen expression in hGFs, and reduced the wound area [14]. Therefore, we decided to investigate the ability of the purple roots of *Lithospermum erythrorhizon*, a natural substance, to promote wound healing. The roots of *L. erythrorhizon* have been used to treat wounds, burns, ulcers, and other conditions since ancient times [15]. Shikonin, an active ingredient of *L. erythrorhizon* roots, has a molecular weight of 288.5 and a chemical formula C_16_H_16_O_5_. Shikonin promotes wound healing [16,17], and exerts anti-inflammatory [18,19], antitumor [20,21], and antimicrobial effects [22]. Furthermore, various pharmacological actions of shikonin have been reported [15,23]. Shikonin formulations currently marketed as medicines include Heriderm^®^ (PNG Gerolymatos, Athens, Greece), Histoplastin Red^®^ (Heremco Pharmaceuticals, Metamorfosi, Greece), Epouloderm^®^ (Farmalex S.A, Athens, Greece), and Shiunko Plaster (for dermatological applications) [23]. These medicines are rarely used to treat wounds, burns, or ulcers in gingival tissue. Papageorgiou reported an 80% success rate for the treatment of chronic leg ulcers with Histoplastin Red^®^, suggesting that this was an effective treatment strategy for this condition [24,25,26]. However, studies specifically focused on the effects of shikonin on gingival tissue wound healing are limited.

Wound healing is a complex process that involves the migration, proliferation, and differentiation of many cell types, the removal of damaged tissue, and the formation of the extracellular matrix [27,28]. The wound-healing process consists of inflammatory, proliferative, and remodeling phases. During the proliferative phase, cells proliferate and migrate, resulting in wound closure. During the remodeling phase, components of the extracellular matrix, such as collagen, are secreted, and the original tissue is regenerated [3]. ERK 1/2 and MAPK are also involved in the wound-healing process. The phosphorylation of ERK 1/2 has been reported in various cell types as an important contributor to cell proliferation, migration, and extracellular matrix formation [29].

The purpose of the present study was to investigate the effects of shikonin on hGFs, which play an important role in the wound healing of gingival tissue.

## 2. Results

### 2.1. Cell Proliferation Assay and LDH Activity

**Figure 1:**
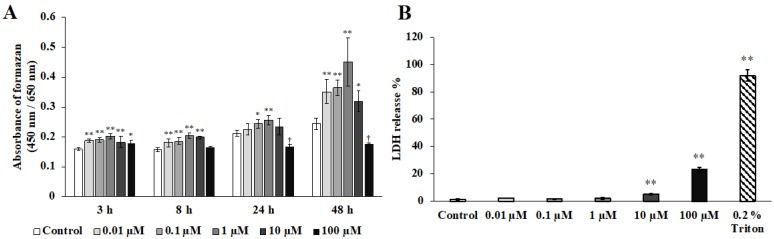
The effect of shikonin on human gingival fibroblast cell proliferation and the cytotoxicity of shikonin. Following cell adherence, the medium was replaced with culture medium, including 10% FBS without (control) or with shikonin (0.01, 0.1 1, 10, or 100 µM). (**A**) Cell proliferation was determined after 3, 8, 24, or 48 h (* *p* < 0.05 at 3, 8, 24, and 48 h vs. control; ** *p* < 0.01 at 3, 8, 24, and 48 h vs. control; ^†^ *p* < 0.01 at 3, 8, 24, and 48 h vs. control). (B) Cytotoxicity was determined after 48 h (** *p* < 0.01 vs. control), with Triton (0.2%) used as the positive control.

Shikonin promoted the growth of hGFs, with the greatest growth observed in response to treatment with 1 μM shikonin (Figure 1A). Treatment with shikonin at 100 μM significantly inhibited cell proliferation. Furthermore, treatment with both 10 and 100 μM shikonin significantly increased LDH activity (Figure 1B).

### 2.2. Cell Migration and Wound Healing Assays

**Figure 2:**
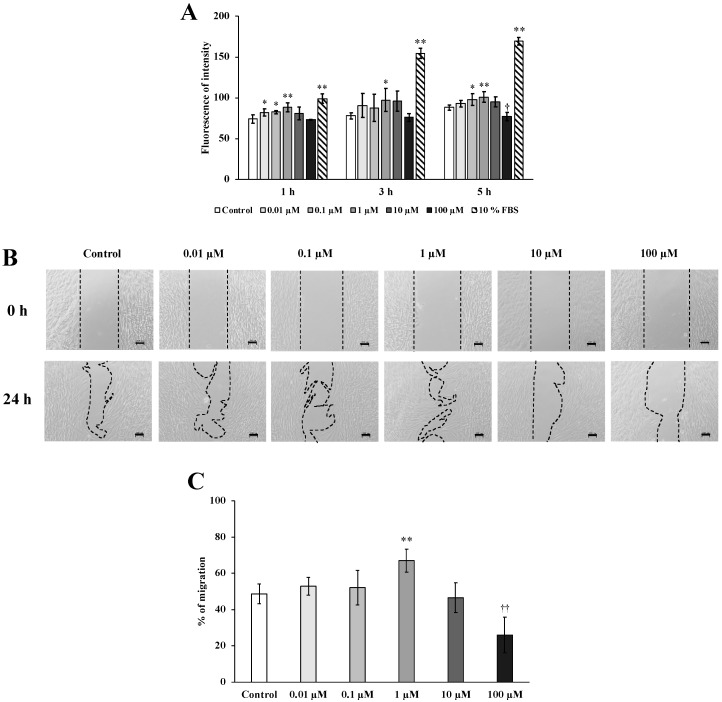
Effect of shikonin on human gingival fibroblast (hGF) migration and wound repair. (**A**) hGF migration in response to control or shikonin treatment (0.01, 0.1, 1, 10, or 100 µM) (* *p* < 0.05 at 1, 3, and 5 h vs. control; ** *p* < 0.01 at 1, 3, and 5 h vs. control; ^†^ *p* < 0.05 at 1, 3, and 5 h vs. control). Fetal bovine serum (10%) was used as the positive control. (**B**) Wound healing was measured after 0 and 24 h (Scale bar = 100 µm). (**C**) The change in the wound area is presented as the ratio of the final to the initial wound size (** *p* < 0.01 vs. control; ^††^ *p* < 0.01 vs. control).

The evaluation of cell migration was performed by using a Boyden chamber (Figure 2A) and a wound-healing assay (Figure 2B,C). Shikonin promoted hGF migration, with the greatest effect observed at 1 μM. In contrast, 100 μM shikonin significantly inhibited cell migration.

### 2.3. Synthesis of Type I Collagen and FN, Gene Expression of VEGF and FN

**Figure 3:**
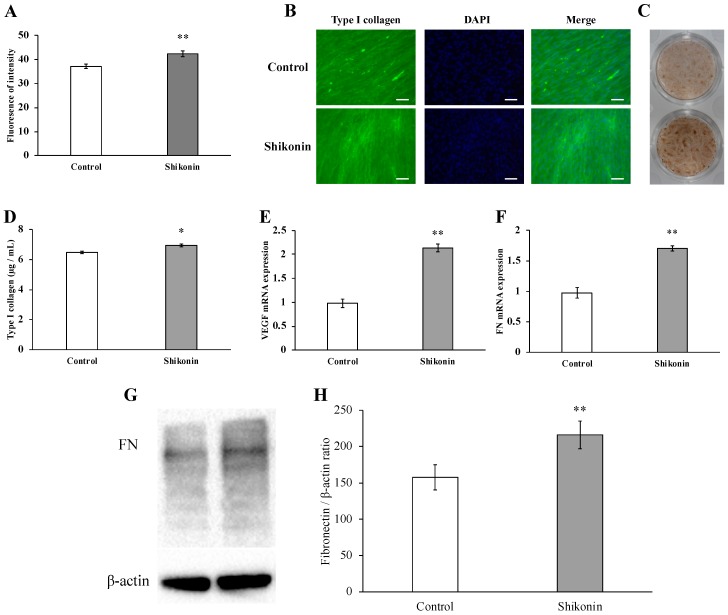
Type I collagen synthesis by human gingival fibroblasts (hGF) treated with or without 1 µM shikonin. Immunostaining for type I collagen using an anti-type I collagen monoclonal antibody. (**A**) Intensity of fluorescence immunostaining without (control) or with shikonin (** *p* < 0.01 vs. control). (**B**) Cells were incubated with fluorescently labeled secondary antibody and stained with DAPI (scale bar= 500 μm). (**C**) After incubation of the cells with the secondary antibody, chromogenic immunostaining was performed, and images were obtained by using a single-lens reflex camera. (**D**) Type I collagen in the cell culture supernatant after treatment for 1 week was measured by using ELISA. (* *p* < 0.05, control vs. 1 µM shikonin). (**E**,**F**) Effects of shikonin on the gene expression of VEGF and FN in stimulated hGFs incubated with 1 µM shikonin for 24 h prior to cell harvest. The gene expression of VEGF and FN was determined by using PCR. (** *p* < 0.01, control vs. 1 µM shikonin). (**G**) Protein expression of FN was evaluated by immunoblotting analysis. (**H**) The quantification was performed using image analysis software. (** *p* < 0.01 vs. control).

We determined the effect of shikonin on hGF type I collagen synthesis. The fluorescence intensity of type I collagen by fluorescent immunostaining was significantly increased in response to treatment with 1 µM shikonin (Figure 3A,B). The intensity of DAB staining of type I collagen was also elevated in response to treatment with 1 µM shikonin (Figure 3C). It was also confirmed that Type I collagen in the cell supernatant was also significantly increased by 1 μM shikonin (Figure 3D). In addition, we showed that the gene expression of VEGF, a growth factor with a major role in wound healing, and FN, a cell adhesion factor, was significantly increased at 24 h after treatment with 1 μM shikonin (Figure 3E,F). We also found to be expressed significantly by the production also 1 μM Shikonin of FN (Figure 3G,H).

### 2.4. Shikonin Activated the ERK 1/2 Signaling Pathway in hGFs

**Figure 4:**
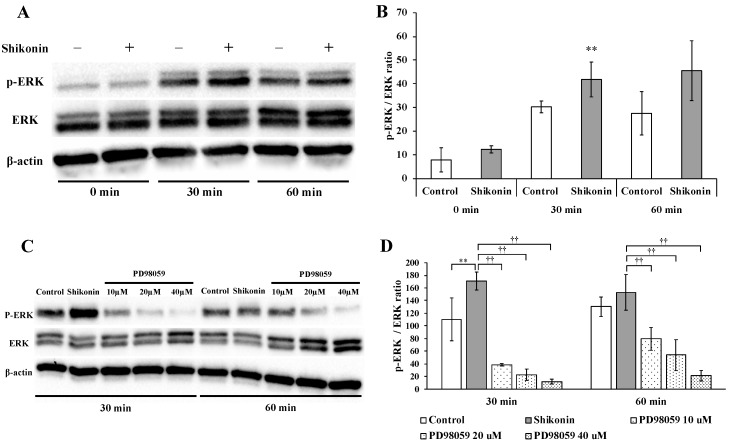
Effect of shikonin on the activation of the MAPK signaling pathway component ERK 1/2. Human gingival fibroblasts were cultured in medium without (control) or with 1 µM shikonin for 0, 30, or 60 min. (**A**) Protein expression was evaluated by immunoblotting analysis. (**B**,**D**) The quantification was performed using image analysis software (** *p* < 0.01, control vs. 1 µM shikonin; ^††^ *p* < 0.01, 1 µM shikonin vs. 1 µM shikonin + PD98059 (10 µM, 20 µM and 40 µM). (**C**) Effect of PD98059 (ERK 1/2 inhibitor) on shikonin-stimulated activation of the MAPK signaling pathway.

To investigate the mechanism of the shikonin-induced proliferation and migration of hGFs, we evaluated the activation of ERK 1/2. The levels of phospho-ERK1/2 in shikonin-stimulated cells were significantly increased in 30 min (Figure 4A,B).

### 2.5. ERK 1/2 Inhibition

**Figure 5:**
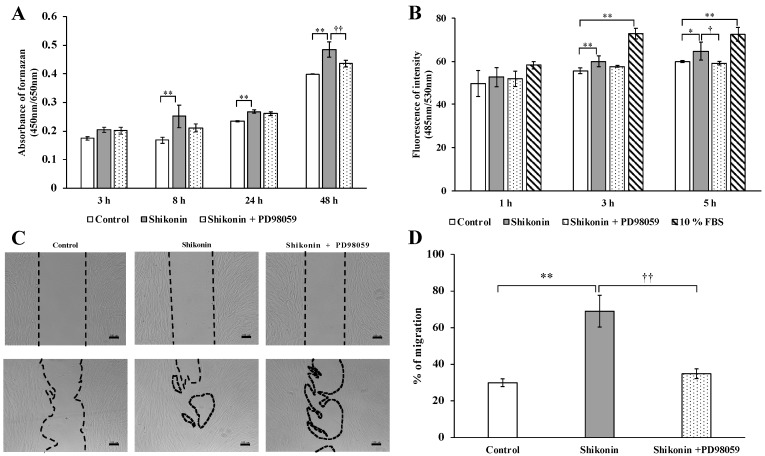
Effects of PD98059 on shikonin-stimulated human gingival fibroblast (hGF) cell proliferation and migration and wound repair. Treatment with 20 µM PD98059 reduced 1 µM shikonin-stimulated hGF (**A**) proliferation and (**B**) migration. (**C**) Images of wound repair and (**D**) quantitative data for wound repair. (* *p* < 0.05, control vs. 1 µM shikonin; ** *p* < 0.01, control vs. 1 µM shikonin; ^†^ *p* < 0.05, 1 µM shikonin vs. 1 µM shikonin + 20 µM PD98059; ^††^ *p* < 0.01, 1 µM shikonin vs. 1 µM shikonin + 20 µM PD98059). In the cell-migration assay, 10% fetal bovine serum was used as a positive control.

**Figure 6:**
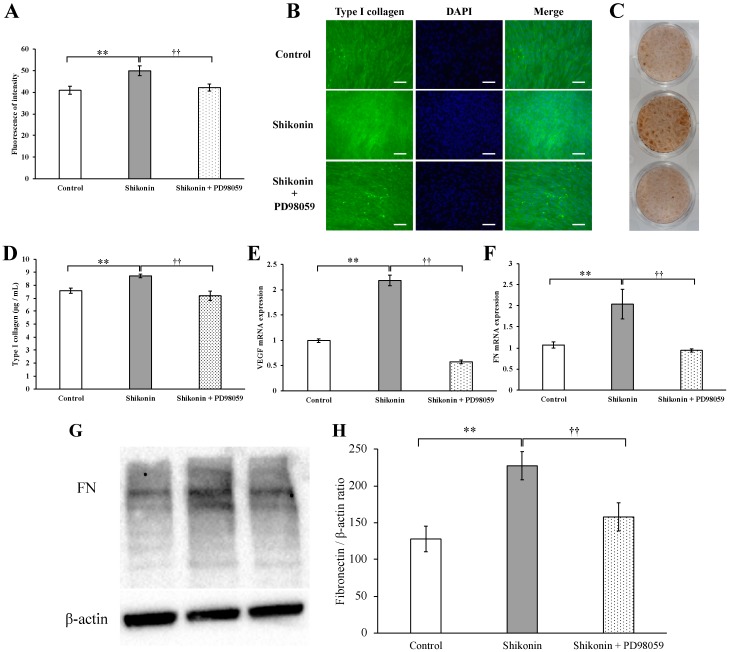
Effect of PD98059 on type I collagen synthesis stimulated by shikonin in human gingival fibroblasts. Type I collagen synthesis was determined by (**A**) fluorescence immunostaining and (**B**) fluorescence microscopy. (scale bar = 500 μm). (**C**) The effect of PD98059 on type I collagen synthesis was also analyzed by chromogenic immunostaining and photographed using a single-lens reflex camera. (**D**) Type I collagen in the cell culture supernatant after treatment for 1 week was measured by using ELISA. (**E**,**F**) Effect of PD98059 on the expression of VEGF and FN stimulated by shikonin in hGFs. Gene expression was determined by PCR. (**G**) Protein expression of FN was evaluated by immunoblotting analysis. (**H**) The quantification was performed using image analysis software. (** *p* < 0.01, control vs. 1 µM shikonin; ^††^ *p* < 0.01, 1 µM shikonin vs. 1 µM shikonin + 20 µM PD98059).

To investigate the role of ERK 1/2 in shikonin-induced proliferation and migration, shikonin-stimulated hGFs were cultured in normal medium containing the ERK 1/2 inhibitor PD98059. Treatment with 10, 20, and 40 μM PD98059 significantly inhibited ERK 1/2 phosphorylation at 30 and 60 min (Figure 4C,D). Furthermore, PD98059 significantly reduced the shikonin-stimulated cell proliferation (Figure 5A) and migration (Figure 5B–D), extracellular matrix, and type I collagen synthesis in the cell supernatant (Figure 6A–D). Treatment with PD98059 significantly reduced the gene expression of VEGF and FN induced by shikonin (Figure 6E,F). We also found PD98059 to be inhibited by the production of FN (Figure 6G,H).

## 3. Discussion

In the present study, shikonin promoted the proliferation and migration of hGFs, the synthesis of the extracellular matrix protein type I collagen and FN, enhanced FN and VEGF gene expression, and the phosphorylation of ERK 1/2. Each of these effects was suppressed by treatment with an ERK 1/2 inhibitor. Previous reports showed that shikonin promoted the proliferation of various normal cells, including skin-derived fibroblasts [30], human epithelial keratinocytes [31,32], and vascular endothelial cells [32]. However, the effects of shikonin on hGFs in wound healing have not previously been reported.

The present study showed that 1 μM shikonin was the most effective concentration for the stimulation of cell proliferation. This result was consistent with reports that shikonin stimulated the proliferation of fibroblasts derived from human skin and human epidermal keratinocytes, and that hydroxylated shikonin (deoxyshikonin) stimulated the proliferation of human umbilical vein endothelial cells [31,32]. In the present study, increased LDH activity was observed in response to treatment at higher shikonin concentrations (10 and 100 μM). This result may have occurred because shikonin is a type of naphthoquinones; these compounds have been reported to induce cell death and act as antitumor compounds [32]. Our results suggested that 1 µM shikonin was the optimal concentration for the evaluation of cell proliferation, whereas higher concentrations (10 and 100 µM) were cytotoxic. Furthermore, our results showed that shikonin-induced cell growth and cytotoxicity were concentration-dependent. Fan et al. reported that 3 µg/mL (10 µM) of shikonin is cytotoxic in human dermal fibroblasts and suppresses cell proliferation [33]. Present study shows that 10 µM shikonin promoted in the cell proliferation assay in the hGF but is cytotoxic in the LDH activity. This result suggests that there is a time and concentration at which the cell proliferative capacity appears depending on the type of cells.

Cell migration was investigated by using a modified Boyden chamber assay and in an in vitro model of wound healing. A previous study showed that the hydroxylated shikonin, deoxyshikonin, promoted human epidermal keratinocyte migration [30]. In the present study, we showed that 1 μM shikonin treatment promoted cell migration. However, 100 μM shikonin treatment suppressed cell migration. These results indicated that the migration of hGFs was induced by treatment with 1 μM shikonin. In the in vitro wound healing model, both proliferation and cell migration can be considered to promote wound closure. This assay has been used to evaluate cell migration in various studies [34,35].

Type I collagen, a major protein in gingival tissue, plays an important role in the healing of connective tissue by providing tissue strength and a scaffold for cell adhesion and migration [3]. Karayannopoulou et al. reported that collagen production was significantly stimulated by a shikonin-containing ointment in a canine model of skin wound healing [36]. In addition, shikonin and its derivatives induced granulation tissue formation and collagen production by fibroblasts in vivo [37]. An increase in inflammatory mediators is known to reduce the expression and synthesis of collagen in fibroblasts, consequently promoting periodontal tissue degradation [38].

Our results demonstrated that shikonin, which exhibits anti-inflammatory properties, significantly enhanced hGF type I collagen synthesis.

Fibronectin can interact with other matrix proteins and macromolecules to promote granulation tissue formation [39]. Furthermore, plasma fibronectin can covalently bind to fibrin to form a fibrotic clot structure that promotes leukocyte and fibroblast migration and adhesion. Fibroblasts subsequently migrate to the wound space and produce more fibronectin, which assembles into a disulfide-bridged temporary matrix. Shikonin has been shown to enhance fibronectin production in human amniotic fibroblasts [40].

Sakaguchi et al. investigated shikonin- and shikonin derivative-induced VEGF production and angiogenesis in mouse granulomatous tissue. Their results indicated that shikonin and its derivatives induced granulomatous tissue formation, and histologic observations showed that a pouch was formed in necrotic tissues directly facing the submuscular connective tissue and cavity, and that granulomatous tissue was generated in the connective tissue. The results showed that extracts of shikonin and shikon (*Lithospermum erythrorhizon* roots) induced angiogenesis in granulomatous tissue [41].

Our study also showed that shikonin enhanced FN and VEGF gene expression and FN production. This finding demonstrated that shikonin promoted wound healing by enhancing angiogenesis and strengthening the adhesion of gingival connective tissue. Several naturally occurring substances are known to induce biological effects through various signaling pathways. For example, lucidone, a component of *Lindera erythrocarpa* Makino, promotes cell proliferation and migration through phosphatidylinositol 3-kinase, Wnt/β-catenin, the epithelial-mesenchymal transition, and nuclear factor-κB/matrix metalloproteinase pathways in keratinocytes and fibroblasts [42]. Previous studies reported that deoxyshikonin, a hydroxide of shikonin, promoted cell proliferation and migration by the activation of ERK 1/2, a MAPK protein, in human umbilical vein endothelial cells [43]. The MAPK signaling pathway is composed of a series of serine/threonine kinases that mediates signaling from the cell surface to the nucleus and includes ERK1/2, c-jun N-terminal kinase (JNK), and p38 [43]. ERK 1/2 is mainly activated by mitogens, whereas JNK and p-38 are preferentially activated by stress-induced stimuli, including UV light, heat shock, and proinflammatory cytokines [44]. The ERK 1/2 signaling pathway has been reported to play a major role in growth factor-induced cell proliferation [29]. The ERK 1/2 signaling pathway is also significantly involved in cell migration and the secretion of extracellular matrix [45,46].

Therefore, we investigated the involvement of the ERK 1/2 signaling pathway in shikonin treatment. In the present study, phosphorylation of ERK 1/2 was enhanced by shikonin treatment during the proliferative phase [27]. We also found that shikonin activated the ERK 1/2 signaling pathway in hGF. PD98059 (20 µM) inhibited shikonin-stimulated hGF proliferation, migration, and type I collagen synthesis. These results suggested that shikonin may promote wound healing via the ERK 1/2 signaling pathway in hGF.

Future studies will focus on the effects of shikonin on wound healing in vivo. This study found that shikonin exerted enhanced cell proliferation, migration, type I collagen and FN synthesis, and the gene expression of VEGF and FN. In addition, we demonstrated that these effects were likely mediated by the ERK 1/2 signaling pathway. This is the first study to evaluate the effects of shikonin on hGFs in the context of wound healing. Therefore, shikonin may have beneficial effects on wound healing after periodontal surgery.

## 4. Materials and Methods

### 4.1. Cell Culture

Human tissue experiments were performed in accordance with the guidelines of the Osaka Dental University for Medical Ethics, and all experiments were approved by the Osaka Dental University Medical Ethics Committee (Approval No. 110989). All participants (three females, between 21 to 27 years of age) provided written informed consent to participate in this study, and the study design was approved by the appropriate ethics review boards.

Human gingival tissue derived from the third molar with non-inflamed gingiva was collected. The gingival tissue was washed in Dulbecco′s Modified Eagle′s Medium (DMEM) (Nacalai Tesque, Kyoto, Japan), cut into 1 mm^3^ pieces, placed on the bottom surface of a 6-well plate, and then suspended in DMEM supplemented with 10% fetal bovine serum (FBS) (Thermo Fisher Scientific, Rockford, IL, USA), 500 U/mL penicillin, 500 U/mL streptomycin, and 0.25 µg/mL amphotericin B (Nacalai Tesque) at 37 °C in a 5% CO_2_ atmosphere. The culture medium was changed every 3 days, and spindle-shaped cell outgrowths from the gingival tissue pieces, which were composed of hGFs, were collected. Human gingival fibroblasts were subcultured to passages 5 and used for each experiment.

### 4.2. Preparation of Shikonin

Shikonin (Nacalai Tesque, the lot no. was Z7F2567, product by Nagara Science Co., Ltd., Gifu, Japan) was dissolved in dimethyl sulfoxide (Nacalai Tesque) and prepared at five concentrations (0.01, 0.1, 1, 10, and 100 μM) for experiments, in addition to a control (0 µM shikonin).

### 4.3. Cell Proliferation Assay

Human gingival fibroblasts were plated in 96-well microplates at 2 × 10^4^ cells/mL in 100 µL normal culture medium. After allowing cells to adhere for 24 h, the medium was replaced with medium containing shikonin (0.01, 0.1, 1, 10, or 100 µM) or without shikonin (control) and the cells were incubated for 3, 8, 24, or 48 h. The number of viable cells at each time point was determined by measuring the amount of formazan generated in four wells per group using Cell Count Reagent SF (Nacalai Tesque). The absorbance of each well at 450 nm was measured, and the data were analyzed by using SoftMax^®^ Pro Microplate Data Acquisition and Analysis software (Version 7.0, Molecular Devices, Sunnyvale, CA, USA).

### 4.4. Cell Cytotoxicity Assay

Human gingival fibroblasts were seeded in 96-well microplates at 2 × 10^4^ cells/mL in 100 μL normal culture medium. The cells were allowed to adhere for 24 h, the medium was exchanged for medium containing shikonin (0.01, 0.1, 1, 10, or 100 μM) or without shikonin (control), and the cells were incubated for 48 h. Triton (0.2%, Sigma-Aldrich, St. Louis, MO, USA) was used as a positive control. Lactate dehydrogenase (LDH) activity was measured using the Cytotoxicity LDH Assay Kit-WST (Dojindo Laboratory, Kumamoto, Japan), as described in the instruction manual. The absorbance of each well was measured at 490 nm, and the data were analyzed by using the SoftMax^®^ Pro Microplate Data Acquisition and Analysis software to determine the formazan content.

### 4.5. Cell Migration Assay

A modified Boyden chamber assay was performed by using 24-well microchemotaxis chambers (Corning Incorporated Life Sciences, Corning, NY, USA) (Fluoroblock^TM^ insert system). Human gingival fibroblasts were incubated with 4 μM Calcein AM solution (Dojindo) for 30 min at 37 °C. The cells were trypsinized, and the reaction was stopped by the addition of a trypsin inhibitor (Nacalai Tesque). The cells were washed with medium and resuspended in serum-free medium to a final concentration of approximately 2.5 × 10^4^ cells/500 μL.

The cell suspension was added to the upper chamber of a cell culture insert, and 750 µL of medium without (control) or with shikonin (0.01, 0.1, 1, 10, or 100 µM) was added to the lower chamber. Medium containing 10% FBS was added to the lower chamber as a positive control. The upper and lower wells were separated by a 3.0 µm pore size HTS Fluoroblock^TM^ Insert (Corning Incorporated Life Sciences). Cell migration was observed after 1, 3, and 5 h. The number of hGFs that passed through the filter to the lower chamber was evaluated by using SoftMax^®^ Pro Microplate Data Acquisition and Analysis software at 485/530 nm excitation/emission [47].

### 4.6. Wound Healing Assay

In vitro wound healing assays were performed by using a wound-repair assay kit (Ibidi GmbH, Am Klopferspitz 19, Martinsried, Germany). Human gingival fibroblasts were seeded at 3.5 × 10^4^ cells/70 µL in a cell culture insert. When the cells reached confluence, the culture insert was lifted, and the medium was replaced with serum-free medium without (control) or with shikonin (0.01, 0.1, 1, 10, or 100 µM). The images of each wound at 0 h and 24 h were collected by using a BZ-II all-in-one fluorescence microscope (Keyence Corporation, Osaka, Japan). The images were used to determine the denuded area by using ImageJ software (National Institutes of Health, Bethesda, MD USA). The data are presented as the percentage of healed wound area at 24 h compared with the initial wound area (0 h) [48].

### 4.7. Gene Expression

Gene expression was assessed by using real-time reverse transcriptase polymerase chain reaction (RT-PCR) (TaqMan; Applied Biosystems, Thermo Fisher Scientific, Waltham, MA, USA). hGFs were seeded in a 24-well microplate at 4 × 10^4^ cells/mL in 1000 μL normal culture medium. After 24 h, the cells were stimulated with either control or 1 μM shikonin. The cells were cultured for 1 week.

Total RNA was isolated by using a kit (RNeasy Mini Kit; Qiagen, Venlo, The Netherlands). RNA (10 μL) from each sample was reverse transcribed to complementary DNA by using a kit (PrimeScript Reagent Kit; Takara Bio, Shiga, Japan).

The gene expression of vascular endothelial growth factor (VEGF) and fibronectin (FN) (Taqman gene expression assay, VEGFA; Hs00900055_m1 and FN1; Hs01549976_m1) was quantified using PCR (StepOne Plus Real-Time PCR System; Applied Biosystems, Thermo Fisher Scientific, Waltham, MA, USA). Using the gene expression results from the negative control group, the ΔΔCt method and normalized to GAPDH was used to calculate the relative gene expression for each group.

### 4.8. Type I Collagen Synthesis

Human gingival fibroblasts were seeded in 24-well microplates at 4 × 10^4^ cells/mL in 1000 μL of normal culture medium. After the cells were allowed to adhere for 24 h, the medium was replaced with control medium or with medium containing shikonin and incubated for 1 week. Following incubation, the cells were fixed with 70% alcohol at −4 °C, washed with PBS (Nacalai Tesque), and permeabilized by the addition of 0.5% Triton X-100 (Sigma-Aldrich) diluted in PBS. Blocking was performed by using 3% bovine serum albumin phosphate buffer solution (Sigma-Aldrich) diluted with PBS and incubated overnight at 4 °C with 500-times diluted primary rabbit anti-human type I collagen antibody (ab 34710) (Abcam, Cambridge, UK).

Fluorescent immunostaining was performed using Alexa Fluor 488^®^ (Thermo Fisher Scientific, Waltham, MA, USA) and the nuclei were stained with 4′,6-diamidino-2-phenylindole (DAPI) (Dojindo). After staining, the images were obtained by using a fluorescence microscope, and the fluorescence intensity was then determined by using SoftMax^®^ Pro Microplate Data Acquisition and Analysis software at excitation/emission of 495/519 nm. The wells were immunostained by using EnVision^TM^ + System-HRP Labeled Polymer Anti-Rabbit (Agilent Technologies, Tokyo, Japan) as the secondary antibody, followed by staining with a chromogenic reagent (DAB Chromogen/Substrate Kit) (Agilent Technologies). The images were collected by using a digital single-lens reflex camera (Nikon, Tokyo, Japan) [32]. Moreover, to measure type I collagen in the cell supernatant after 1 week of culture, the measurement was performed in accordance with the instructions by using the human collagen type I ELISA kit (ACEL Inc., Kanagawa, Japan).

### 4.9. Western Blots

Human gingival fibroblasts were treated with 1 μM shikonin for 0, 30, or 60 min. The total protein was extracted by using RIPA buffer (Thermo Fisher Scientific, Waltham, MA, USA) supplemented with a protease inhibitor cocktail (Thermo Fisher Scientific, Waltham, MA, USA). The total protein concentration was determined by using a BCA Protein Assay kit (Thermo FisherScientific, Waltham, MA, USA).

Protein samples were separated by 12.5% SDS-PAGE (Nacalai Tesque) and transferred onto polyvinylidene difluoride membranes (Bio Rad, Hercules, CA, USA). Non-specific binding to the membranes were blocked by using Blocking One (Nacalai Tesque), and the membranes were incubated overnight at 4 °C with primary antibodies to ERK and phospho-ERK (Cell Signaling Technology, Danvers, MA, USA). The membranes were washed with 0.1% Tween 20—TBS (10×) (pH 7.4) tested for nucleases and proteases (Nacalai Tesque), and then incubated with secondary antibodies for 3 h at room temperature. Immunoreactive bands were visualized by using a chemiluminescence kit (Nacalai Tesque); the signals and western blotting data were analyzed by using the ChemiDoc MP System (BioRad) [49]. The statistical analyses were performed using Image Lab software (Version 5.0, BioRad).

hGF were treated 1 µM Shikonin for 1week and assayed in the same manner as described above. Anti-Fibronectin antibody (ab 2431) was used as the antibody (Abcam).

### 4.10. ERK 1/2 Inhibition

To determine the optimal concentration of the ERK_1/2 inhibitor PD98059 (ChemScene LLC, Monmouth Junction, NJ, USA), western blotting was performed on cells treated with PD98059 at three concentrations (10, 20, and 40 μM). The western blotting data were quantified by using Image Lab software (Version 5.0, BioRad, Hercules, CA, USA). After the optimal concentration was determined, cell proliferation and migration, and immunostaining for type I collagen were compared in response to treatment with shikonin with or without PD98059.

### 4.11. Statistical Analyses

Statistical analyses were performed by using IBM SPSS Statistics Ver. 17 (IBM, Chicago, IL, USA). One-way analysis of variance, followed by Tukey’s post-hoc test, was used to determine significance. *p*-values < 0.05 were considered significant. In this study, all experiments were performed in triplicate.

## 5. Conclusions

The findings of this study suggest that shikonin promotes hGF proliferation, migration, type I collagen and FN synthesis, and the gene expression of VEGF and FN via ERK 1/2 signaling.
